# Socio-motivational moderators—two sides of the same coin? Testing the potential buffering role of socio-motivational relationships on achievement drive and test anxiety among German and Canadian secondary school students

**DOI:** 10.3389/fpsyg.2015.01675

**Published:** 2015-10-31

**Authors:** Frances Hoferichter, Diana Raufelder, Michael Eid

**Affiliations:** ^1^Department of Education and Psychology, Free University of Berlin, Berlin, Germany; ^2^Department of Educational Science, Ernst-Moritz-Arndt-Universität Greifswald, Greifswald, Germany

**Keywords:** achievement drive, German and Canadian secondary school students, multigroup latent moderated structural equations, socio-motivational relationships, test anxiety

## Abstract

The current cross-national study investigates the potential buffering role of socio-motivational relationships for the association of achievement drive (AD) and test anxiety (TX) in secondary school students from Canada and Germany. One thousand and eighty-eight students (54% girls, *M*_age_ = 13.71, SD = 0.53, age span 12–15 years) from the state of Brandenburg and 389 students from Quebéc (55.9% girls, *M*_age_ = 13.43, SD = 0.82, age span 12–16 years) were asked about their socio-motivational relationships with their teachers and peers, their drive for achievement, and TX. Multigroup latent moderated structural equations were conducted to test for the moderator role of socio-motivational relationships that would buffer feelings of TX related to the drive for achievement. The analyses revealed the two-sided role socio-motivational relationships can have for students with different levels of AD; intensifying or mitigating feelings of TX. Thereby, the results of this study extend the buffering hypothesis by Cohen and Wills (1985). Cross-national differences between Canada and Germany were found concerning the studied moderators on the association of AD and TX: While for German students teacher–student relationships acted as moderator, for Canadian students student–student relationships and teachers acting as positive motivators displayed a moderator role.

## Introduction

The drive for achievement has been found to be a basic human need which includes the individual’s desire to successfully accomplish challenges, succeed in competitions, and excel in activities evaluated as important (Atkinson, 1957; McClelland et al., 1976; Covington, 1992). Achievement drive (AD) has been defined in many different ways, yet it is usually described as a mentality by which individuals compare themselves and their performances to the standards of others against whom they stand in competition (Singh, 2011). It has also been understood as a combination of personality traits related to successful performances and/or failure-avoiding behavior (Atkinson, 1974) and has been conceptualized as task-oriented behavior (Nicholls, 1984; Ames, 1992), constituting a learned drive (Atkinson, 1957; McClelland, 1961). Based on the manifold definitions regarding AD, the current study views the drive for achievement as a multifaceted construct.

### Achievement Drive in the Western World

In the context of education, achievement is actively promoted as an absolute necessity and it is given high priority (Kaplan and Maehr, 2007). This notion is accompanied by teachers’ beliefs and instructional practices, which are mainly oriented toward mainstream cultural themes such as competition and individualism (Boykin et al., 2005; Tyler et al., 2006). Besides academic goals such as learning, improving performance, demonstrating ability, and outperforming others, social goals such as enhancing a sense of belonging, obtaining social approval by peers, teachers, parents, and gaining tangible rewards for academic performances were identified as the main rationale for students’ AD (Dowson and McInerney, 2003; Levy et al., 2004; Hoferichter and Raufelder, 2014).

On a behavioral and cognitive level, the drive for achievement has been shown to play a divergent role: on the one hand, it is a prerequisite for successful learning, performance, and adaptive behavior (Wang et al., 1993; Pintrich and Schunk, 1996; Weinstein, 1998; Steinmayr and Spinath, 2008) and is positively related to students’ persistence, academic efficacy, positive attitudes toward school, school engagement, and a positive school self-concept (Eccles et al., 1998; Elliot, 1999; Harackiewicz et al., 2002; March and Craven, 2005; for a review, see Urdan, 1997). On the other hand, some problematic outcomes have been found to be positively related to AD, for example behavior of avoidance, anxiety, disruptive behavior, low retention of knowledge, and the use of superficial learning strategies (Covington, 1992; Selkirk et al., 2011), which are also elements of test anxiety (TX; Zeidner, 1998; Frydenberg, 2002; Cortina, 2008). Hence, students who strive for academic achievement are eager to avoid failure and may feel constant pressure to perform well (cf. Atkinson, 1957; Midgley et al., 2001). Adding to these results, Pekrun (2000) found that achievement related anxiety was the most frequently reported emotion among school and university students.

### Achievement Drive and Test Anxiety in the School Context

Studies on academic motivation stress the importance of students’ social environments (Pintrich and Schunk, 1996; Eccles and Roeser, 2011), as their motivation emerges from interactions within the school context, especially in the classroom (Ames, 1992; Urdan and Schoenfelder, 2006). With increasing grade level, students’ academic performance and competition with classmates enter the spotlight (Wigfield et al., 1991; Midgley, 1993), while they become increasingly aware of their own abilities and thus familiar with the concept of achievement (Nicholls and Miller, 1983; Nicholls, 1984). As a consequence, students’ desire to succeed and avoid failure catapults some into a profound emotional conflict and ultimately tempts them to cheat (Schab, 1991; Anderman et al., 1998), avoid novelty, and challenges (Gheen and Midgley, 1999). Students’ fear of publicly revealing their incompetence poses a major threat to their person (Schwarzer, 2000) by inducing feelings of shame and insecurity, which often intensify TX (Covington and Omelich, 1988; Covington, 1992; Elliot and McGregor, 1999; Elliot et al., 1999). TX is a multi-faceted personality trait which consists of cognitive and affective components (Hoferichter et al., 2015), including the lack of self-confidence, worrying thoughts about individual performance and the consequences of failure as well as emotional and physical tension (Hodapp and Benson, 1997; Meijer, 2001; Lowe et al., 2011), which overall may impair performance in achievement situations (Zeidner, 1998). Alongside personal characteristics (Schwarzer, 2000; Komarraju et al., 2011; Hoferichter and Raufelder, 2013) and previous negative experiences with evaluations (Pekrun, 2001), the occurrence and development of TX is essentially influenced by peers and teachers (Harter, 1996; Grills and Ollendick, 2002; Biggs et al., 2010; Hoferichter et al., 2014a).

### Environmental Factors: Socio-Motivational Relationships with Peers and Teachers

 Additionally, researchers found that students’ perception of the school context and its various aspects influences their motivation to achieve as well as their TX. In particular, supportive teachers and peers have been found to strengthen students’ motivation (Wang and Eccles, 2013), grade point average, school adjustment, well-being, and lead to a decrease in TX levels as well as dropout rates from school (Buhs et al., 2006; Perry et al., 2007; Rudasill et al., 2010; Wentzel et al., 2010; Hoferichter and Raufelder, 2013). In particular, studies have shown that positive relationships and feelings of belonging to a school context function as buffer and therefore protect individuals in stressful situations, for example during negative experiences in school and in risky situations (Cohen and Wills, 1985; Stravynski and Boyer, 2001; Adams et al., 2011; Johnson et al., 2011; Cavanaugh and Buehler, 2015). Following this premise, Cohen and Wills (1985) formulated the buffering hypotheses, which postulates that relationships perceived as supportive, for example with classmates in college, friends, and co-workers, buffer feelings of stress. Feeling part of a larger dependable and stable structure with which one shares commonalities and socio-emotional ties can be defined as a sense of belonging or connectedness (Sarason, 1974; Unger and Wandesman, 1985). The fundamental need to connect with others is a basic motivating principle of humankind (Smith and Mackie, 2000), which drives the individual to initiate and maintain social relationships and secures mental as well as physical health (Resnick et al., 1993; Adler and Brett, 1998). In this sense, it has been found that people who feel connected with others report higher self-esteem and are less stressed and anxious in comparison to individuals, who report less connectedness (Lee and Robbins, 1998; Lee et al., 2002).

Additionally, cross national studies have shown that peers and teachers are not only perceived as supportive and trustworthy individuals on an interpersonal level but are also viewed as a source of motivation, which students depend on for their own academic achievement (Raufelder et al., 2013b; Hoferichter et al., 2014b). Further research in this field suggests that when students perceive their peers and teachers as motivators, they tend to experience higher TX as they try to meet the expectations of peers and teachers (Hoferichter et al., 2014a). Students also tend to avoid challenges and novelty when their teacher emphasizes the importance of demonstrating ability in the classroom (Gheen and Midgley, 1999). In this sense, students with dependent teacher or peer relationships tend to face behavioral and social problems at school and show poor academic performance (Pianta and Nimetz, 1991; Hamre and Pianta, 2001; Sabol and Pianta, 2012).

### Cross-National Findings

Although the concepts of AD and TX have received much international scholarly attention (Bodas and Ollendick, 2005; Areepattamannil, 2012; De Castella et al., 2013), both national and cross-national research has predominantly taken a narrow approach that focuses on individuals and regrettably neglects contextual variables, which may vary across countries (Markus and Kitayama, 1991; Bodas and Ollendick, 2005), as for example the impact of peers and teachers on students’ AD and TX (cf. Shernoff and Schmidt, 2008). The educational setting wherein students learn and develop their personality and individual competences has an incisive impact on motivation, social competence, and relationships with peers and teachers (Salili et al., 2001; Hurrelmann, 2006; Bünger and Raufelder, 2014; Hoferichter et al., 2014a). For example, while Germany and Canada are both considered to be individualistic societies (Triandis, 1995) and as members of the Organization for Economic Co-operation and Development (OECD), both are committed to certain economic and educational goals, the transfer of knowledge and the scope for and maintenance of social relationships in school vary considerably between these two countries (for an overview, see Hoferichter et al., 2014a). For example, German students report a competitive classroom environment (Graudenz and Randoll, 1997) and generally perceive the scholastic performance of their peers as an incentive for their own performance (Kaufmann, 2007; Raufelder et al., 2013a). The relationship between students and their teachers is generally loose and impersonal as the emphasis of German schools lies on the transfer of knowledge (Graudenz and Randoll, 1997; Beckmann, 2000; Hesse, 2004). In contrast, Canadian schools stress the development and maintenance of social skills, for example with peers and teachers, which consequently helps build a supportive scholastic environment (Wilson and Lam, 2004). One particular cross-national study found that Canadian students especially value social support from friends at school, which was related to high levels of intrinsic achievement motivation (Vitoroulis et al., 2012).

Given the scarcity of cross-national studies on the interplay between AD and TX within the field of education and psychology (Midgley et al., 2001; Stankov, 2010), we address this gap by considering both Canadian and German secondary school students. According to Kinga and McInerney (2014), although culture influences basic motivational processes, cross-national differences have barely been considered and the need to “culturalize educational psychology” is still present (Pajares, 2007). Moreover, in light of the buffering hypothesis (Cohen and Wills, 1985), we investigated whether socio-motivational relationships, such as teacher–student relationships (TSR), student–student relationships (SSR), teachers acting as positive motivators, and peers acting as positive motivators, can function as moderators in the relationship between AD and TX. In other words: Do socio-motivational relationships buffer feelings of TX related to the drive for achievement?

#### Hypothesis 1

We hypothesize that social relationships with peers and teachers moderate the association between AD and TX among Canadian and German secondary school students.

#### Hypothesis 2

We hypothesize that motivational relationships with peers and teachers moderate the association between AD and TX across the two samples.

## Materials and Methods

### Participants and Procedure

During the school year of 2011/2012 and 2012/2013, a total of 1477 seventh and eighth grade students from Germany and Canada participated in the current study. One thousand and eighty-eight German secondary school students (54% girls, 46% boys; *M*_age_ = 13.71, SD = 0.53, age span 12–15 years) from the state of Brandenburg evaluated a set of given statements addressing their socio-motivational relationships with peers and teachers, TX, and AD. The schools were selected at random, after the governmental Department of Education, Youth, and Sport for Brandenburg gave permission to conduct the study. Since German law prohibits obtaining information about a third party’s socio-economic status (SES), we could not ask our participants about their parents’ or guardians’ financial situation or educational background.

Alongside the German participants, 389 secondary school students from Quebéc also participated in the study (56% girls, 44% boys; *M*_age_ = 13.43, SD = 0.82, age span 12–16 years). Various institutions authorized the questionnaire: the ethic commission of Concordia University, the English Montréal School Board (EMSB) and the governing board of each participating school. The Canadian students answered the same questionnaire as the German students, albeit in English.

In both countries, parental permission was obtained and students were informed that the survey was anonymous, confidential, and that their participation was entirely voluntary. Data collection took place on two consecutive days in classrooms or in the cafeteria.

### Measures

#### Achievement Drive

This subscale is part of the questionnaire Achievement Motivation for students (Petermann and Winkel, 2007). The scale consists of eight items and features a reliability of α = 0.82 for the German sample and α = 0.81 for the Canadian sample. Examples of the questions posed to students include: “I make an effort so that my performance is better than the average” and “I prefer to work on tasks that challenge me.” Their answers were classified on a 5-point Likert scale ranging from 1 (*not true at all*) to 5 (*absolutely true*).

#### Test Anxiety

This subscale is also part of the questionnaire Achievement Motivation for students (Petermann and Winkel, 2007). The scale consists of four items and has a reliability of α = 0.72 for the German sample and α = 0.71 for the Canadian sample. Questions include “I get very nervous before exams” and “I am afraid to fail when I have to solve a difficult task,” while answers were classified on a 5-point Likert scale ranging from 1 (*not true at all*) to 5 (*absolutely true*).

#### Peers as Positive Motivators (PPM)

This subscale is part of the relationships and motivation (REMO) scale (Raufelder et al., 2013a) and consists of nine statements such as “When my friends learn, I am also motivated to learn more” or “My friends and I motivate each other to make an effort at school.” This subscale has a reliability of α = 0.80 for German students and α = 0.83 for Canadian students. Students answered questions on a 4-point Likert scale from 1 (*strongly disagree*) to 4 (*strongly agree*).

#### Teachers as Positive Motivators (TPM)

This subscale was also taken from the REMO scale (Raufelder et al., 2013a) and consists of six items with a reliability of α = 0.78 for German students and α = 0.76 for Canadians. Students were asked to answer statements such as “I will make more of an effort in a subject when I think the teacher believes in me” or “When a teacher helps me, I try to do well in the subject.” The answers to the questions ranged from 1 (*strongly disagree*) to 4 (*strongly agree*) on a 4-point Likert scale.

#### Student–Student Relationships

This measure assesses a sense of belonging and the inclusion into the class structure. The instrument was borrowed from the Programme for International Student Assessment (PISA; Kunter et al., 2002) and has an internal reliability of α = 0.70 for German students and α = 0.72 for Canadians. Students rated six statements such as “In our class there are some students that receive little attention from others” and “You easily become an outsider when you are not doing what the class believes to be right” on a 4-point Likert scale from 1 (*strongly disagree*) to 4 (*strongly agree*). The negative items were converted for the current analyses. Thus, high quality SSR describe a tendency to deny negative group behavior such as exclusion, while low quality SSR encode the perception of an excluding class setting, hence a problematic interaction between individuals and groups.

#### Teacher–Student Relationships

This scale was also borrowed from the PISA (Kunter et al., 2002) and has an internal reliability of α = 0.78 for the German sample and α = 0.83 for the Canadian sample. On a 4-point Likert scale from 1 (*strongly disagree*) to 4 (*strongly agree*) students were asked to rate five statements such as “Most of the teachers treat me fairly” and “When I need additional help, I get it from my teachers.”

### Statistical Analyses

First, two models were conducted on the basis of item solutions and not yet parcels. Accordingly, the fit statistic for the model including SSR and TSR was as follows: CFI: 0.90, TFI: 0.90, SRMR: 0.06, RMSEA: 0.05 (0.05–0.05), while the fit statistic for the model including TPM and PPM was as follows: CFI: 0.90, TFI: 0.90, SRMR: 0.06, RMSEA: 0.04 (0.04–0.05). However, after carefully considering the advantages and disadvantages of parceling, we decided to work with parcels, rather than with item based solutions. Working with parcels has become a common procedure in research applying structural equation models (for a review, see Bandalos and Finney, 2001), although it has also been controversially discussed (Marsh et al., 2013), as it may camouflage misspecification with item parcels in confirmatory factor analysis models. Nonetheless, the application of parcels has been shown to result in better fitting solutions and less bias in estimates of structural parameters in comparison to item based solutions (Bandalos, 2002; Little et al., 2002; Nasser and Wisenbaker, 2003). Little et al. (2002) list various reasons, why working with parcels can be advantageous: (1) spurious correlations may be a result of estimating large numbers of items, (2) specific sources of variance that may not be from primary interest may be shared by a subset of items from a large item pool, (3) stable solutions are less likely to be a result of item-level data. Hence, by applying the technique of parceling, the original large number of items is being reduced, yielding stable solutions by preventing potential spurious correlations and variance sharing. Additionally, in their simulation study, Nasser and Wisenbaker (2003) advise using parcels over item solutions, if the sample size exceeds 100, which is the case in the current study.

Subsequently, to test our hypotheses, parcels were built randomly from the scales PPM, TPM, SSR, TSR, AD, and TX to ensure that all measurement information would enter the multigroup moderated structural equations. Building parcels randomly is a common technique in psychological research (Prats, 1990; Marsh et al., 1998; Nasser and Wisenbaker, 2003). Subsequently, all items of the measures were transformed randomly into two parcels each. Hence, the nine items from the PPM scale were transformed into two parcels consisting of four and five items each (PPMP1, PPMP2). The six items of the TPM scale were also transformed into two parcels consisting each of three items (TPMP1, TPMP2), the six items of the SSR scale were transformed into two parcels consisting of three items each (SSRP1, SSRP2), the items of the TSR scale were transformed into two parcels consisting of three and two items each (TSRP1, TSRP2). Furthermore, the eight items of the AD scale were transformed into two parcels with each four items (ADP1, ADP2), and the four items of the TX scale were transformed into two parcels with each two items (TXP1, TXP2).

### Multigroup Latent Moderated Structural Equations

Multigroup latent moderated structural equation (MGLMS) models in Mplus 7 (Muthén and Muthén, 1998–2013) were used to test the hypothesized relations between all variables of interest and to investigate latent interaction effects across the German and Canadian samples. In particular, two MGLMS were conducted to test (1) the moderating role of SSR and TSR as well as (2) the moderating role of PPM and TPM for the association of AD and TX among German and Canadian students using a step-wise latent moderated structural equations (LMS) technique (Klein and Moosbrugger, 2000) respectively.

Latent moderated structural equations represent an extension of ordinary structural equation models (SEM), as they explicitly take into account the non-normality caused by the latent non-linear interaction terms (Klein and Moosbrugger, 2000). Simulation studies have proven that LMS provide efficient parameter estimators and a reliable model difference test, showing no indication of bias of standard errors (Klein, 2000; Klein and Muthén, 2007; Moosbrugger et al., 2009).

As no perfect fit statistic can be obtained for models that include latent interactions, three models without interaction terms were considered first in order to determine the model fit of the corresponding measurement model: Measurement invariance was tested by comparing an (1) unrestricted measurement model with a (2) weak measurement model by using the *χ*^2^-difference test (Satorra and Bentler, 2001). In the unrestricted measurement model, factor loadings and intercepts are free with no restriction whatsoever, i.e., the factor loadings and the intercepts may differ across both countries. In the weak measurement model, equal factor loadings but free intercepts among German and Canadian students are assumed. A non-significant *χ*^2^-difference indicates measurement invariance, rendering a comparison between the two country samples feasible.

Subsequently, the weak measurement model with group differences between the two nations was compared with a (3) strong measurement model without group differences by using the *χ*^2^-difference test (Satorra and Bentler, 2001). The strong measurement model enforces equal factor loadings and intercepts across both groups of students. A significant *χ*^2^-difference test indicates pronounced group differences such that (2) the weak measurement model describes the data better than the strong measurement model does and can thus be used for further analysis.

The model fit was estimated in Mplus using four primary fit indices as recommended by Hu and Bentler (1999): chi-square test of model fit (*χ*^2^), root mean square error of approximation (RMSEA), comparative fit index (CFI), and Tucker-Lewis index (TLI).

Finally, an extended model was derived from the best identified model (unrestricted, weak, or strong measurement model) through the addition of interaction terms. In order to determine which model would fit the data best—the model with interaction terms or the model without interaction terms—both models were compared by means of a log-likelihood difference test (Satorra and Bentler, 2001; Geiser, 2012).

## Results

### Model (A)—Student–Student Relationships/Teacher–Student Relationships

In order to determine which model had the best data fit, the unrestricted model [*χ*^2^(24) = 36.10, *p* < 0.001, CFI = 0.99, TLI = 0.98, SRMR = 0.02, RMSEA (90% CIs) = 0.03 (0.01–0.04)] was compared to the weak measurement model [*χ*^2^(28) = 37.36, *p* < 0.001, CFI = 0.99, TLI = 0.99, SRMR = 0.02, RMSEA (90% CIs) = 0.02 (0.01–0.04)], which was then compared to the strong measurement model [*χ*^2^(32) = 81.21, *p* < 0.001, CFI = 0.97, TLI = 0.95, SRMR = 0.04, RMSEA (90% CIs) = 0.05 (0.03–0.06)] by running a chi-square difference test. When comparing the unrestricted model with the weak measurement model, the *χ*^2^-difference test did not reach a level of significance [*χ*^2^(4) = 1.62, *p* = 0.81], which confirms weak measurement invariance. When comparing the weak measurement model with the strong measurement model, the *χ*^2^-difference test yielded a significance level [*χ*^2^(4) = 52.52, *p* < 0.001], suggesting that the strong measurement model fits the data significantly worse than the weak measurement model. In other words, we can confirm weak measurement invariance and are therefore able to draw conclusions about the association of variables across Germany and Canada (Yuan and Bentler, 2004; Geiser, 2012).

### Model (B)—Peers as Positive Motivators/Teachers as Positive Motivators

Similar analyses were conducted for model (B), comparing the unrestricted measurement model [*χ*^2^(24) = 52.05, *p* < 0.001, CFI = 0.99, TLI = 0.98, SRMR = 0.02, RMSEA (90% CIs) = 0.04 (0.03–0.05)] to the weak measurement model [*χ*^2^(28) = 59.28, *p* < 0.001, CFI = 0.99, TLI = 0.98, SRMR = 0.03, RMSEA (90% CIs) = 0.04 (0.03–0.05)]. When doing so, the difference test did not reach a level of significance [*χ*^2^(4) = 7.432, *p* = 0.115], thus confirming weak measurement invariance. Furthermore, the weak measurement model was compared to the strong measurement model [*χ*^2^(32) = 97.20, *p* < 0.001, CFI = 0.98, TLI = 0.96, SRMR = 0.04, RMSEA (90% CIs) = 0.05 (0.04–0.06)], by running a chi-square difference test to identify any essential cross-national differences. By comparing the weak measurement model with the strong measurement model, the difference test revealed a significant difference [*χ*^2^(4) = 41.05, *p* < 0.001], meaning that the strong measurement model fits the data worse than the weak measurement model does. Consequently, we are able to draw conclusions about the association of variables across Germany and Canada.

### Multigroup Latent Moderated Structural Equation 1 (MGLMS 1 with SSR and TSR as Moderators)

Interaction terms were added to the weak measurement model to test the hypothesis that SSR and TSR moderate the association between AD and TX. Consequently, the weak measurement model without interaction terms was compared with the weak measurement model with interaction terms by applying a log-likelihood difference test to evaluate which model would fit the data best. The difference test revealed a better data fit for the model with interaction terms (MGLMS 1) than for the model without interaction terms [*χ*^2^(4) = 20.83, *p* < 0.001].

**FIGURE 1:**
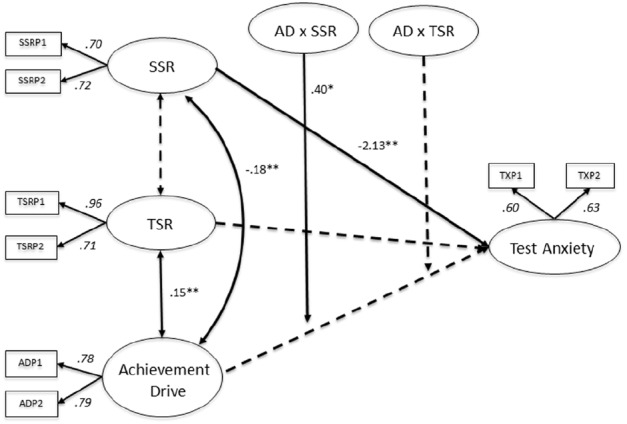
**Multigroup latent moderated structural equations (MGLMS) for the Canadian sample.** Significant effects are shown as unstandardized coefficients (B), bold pathways are significant at *p < 0.05, **p < 0.001; dotted pathways are not significant. Factor loadings are standardized (based on the standardized results of the weak measurement model seen as the final model only reports unstandardized results). TSR, teacher–student relationships; SSR, student–student relationships; AD, achievement drive.

For Canadian students, SSR moderate the association between AD and TX (*B* = 0.40, SE = 0.13, *p* < 0.05), whereas TSR were not identified as a significant moderator. Additional direct effects can be derived from the MGLMS 1 (Figure 1). Among Canadian students, the association between SSR and TX is significant (*B* = –2.13, SE = 0.50, *p* < 0.001). Hence, the better students perceive their relationship with other students to be, the less they report feelings of TX.

**FIGURE 2:**
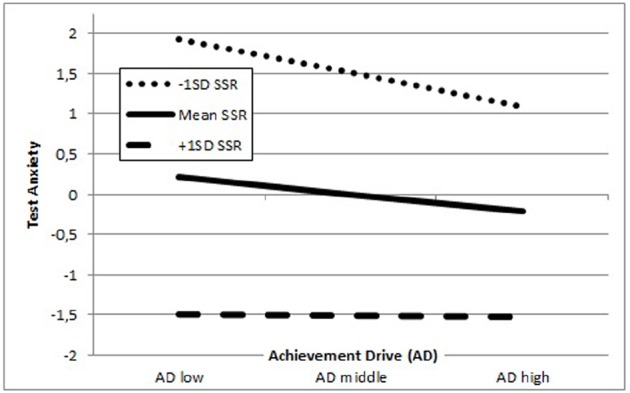
**Representation of student–student relationships (SSR) as moderator in the association of achievement drive (AD) and test anxiety (TX) for Canadian secondary school students; the Y-axis measures test anxiety and the X-axis indicates achievement drive**.

As Figure 2 illustrates, students with rather low quality SSR tend to report high levels of TX over the whole range of AD, with TX decreasing with increasing AD. In contrast, students with rather high SSR report substantially lower levels of TX. In this case TX practically does not vary with changing AD any more. Hence, the perception of high quality SSR buffers feelings of TX in students over the entire range of AD. In turn, students who report rather low quality relationships with their peers start off with higher levels of TX, which decrease with increasing AD but remain clearly above TX levels of students with higher SSR. In conclusion, SSR buffer the association of AD and TX for Canadian students, independent of their levels of AD.

**FIGURE 3:**
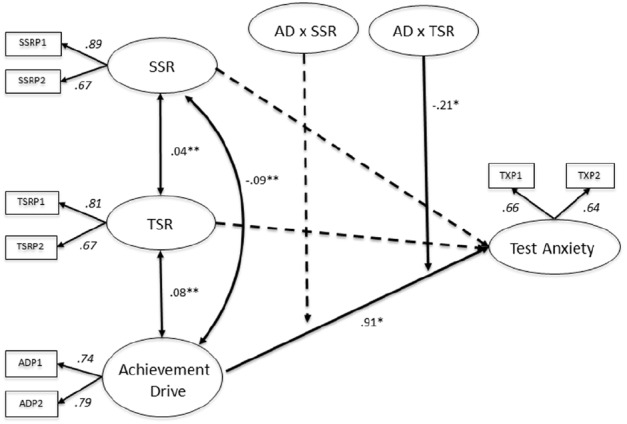
**Multigroup latent moderated structural equations (MGLMS) for the German sample.** Significant effects shown as unstandardized coefficients (B), bold pathways are significant at *p < 0.05, **p < 0.001; dotted pathways are not significant. Factor loadings are standardized (based on the standardized results of the weak measurement model seen as the final model only reports unstandardized results). TSR, teacher–student relationships; SSR, student–student relationships; AD, achievement drive.

When investigating the moderating role of social relationships among German secondary school students, TSR emerged as moderator (*B* = –0.21, SE = 0.11, *p* < 0.05), while SSR did not moderate the association between AD and TX. Additional direct effects can be derived from the MGLMS 1 for German secondary school students (see Figure 3). In detail, AD is positively related to TX (*B* = 0.91, SE = 0.46, *p* < 0.05), indicating that students oriented to achieve also show higher levels of TX.

**FIGURE 4:**
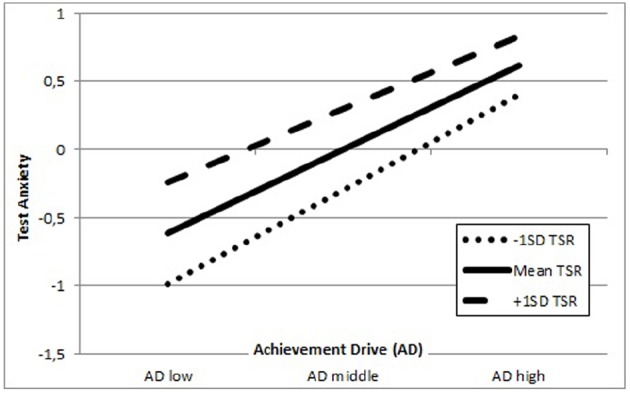
**Representation of teacher–student relationships (TSR) as moderator in the association between achievement drive (AD) and test anxiety (TX) among German secondary school students; the Y-axis measures test anxiety and the X-axis indicates achievement drive**.

The results depicted in Figure 4 indicate that German secondary school students who report low AD and high quality TSR experience increased TX compared to students who have a low AD and low quality TSR. With increasing AD the reported levels of TX increase, irrespective of the quality of TSR, while the slopes of all student groups are similar. In summary, TSR act as moderator in the association between AD and TX, however it cannot be interpreted as a buffer.

### Multigroup Latent Moderated Structural Equation 2 (MGLMS 2 with PPM and TPM as Moderators)

The hypothesis that PPM and TPM would moderate the association between AD and TX (MGLMS 2) among German and Canadian secondary school students was tested, following a similar procedure to that used for the MGLMS 1: interaction terms were added to the weak measurement model (MGLMS 2), which was compared to a model without interaction terms using the log-likelihood difference test to evaluate which model would fit the data best. Having reached a level of significance [*χ*^2^(4) = 11.93, *p* < 0.05] the model with interaction terms (MGLMS 2) was favored.

**FIGURE 5:**
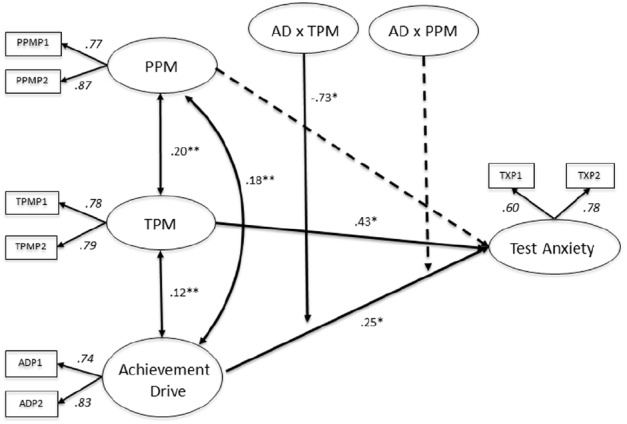
**Multigroup latent moderated structural equations (MGLMS) for the Canadian sample.** Significant effects shown as unstandardized coefficients (B), bold pathways are significant at *p < 0.05, **p < 0.001; dotted pathways are not significant. Factor loadings are standardized (based on the standardized results of the weak measurement model seen as the final model only reports unstandardized results). PPM, peers as positive motivators; TPM, teachers as positive motivators; AD, achievement drive.

Upon examining the Canadian data for potential moderators, TPM were found to moderate the association between AD and TX (*B* = –0.73, SE = 0.28, *p* < 0.05). Furthermore, the MGLMS 2 revealed significant direct effects (Figure 5). For Canadian students, AD is related to TX (*B* = 0.25, SE = 0.12, *p* < 0.05), indicating that the more students are oriented toward achievement, the more they report of TX. Additionally, TPM and TX are significantly related (*B* = 0.43, SE = 0.21, *p* < 0.05). Hence, students who depend on teachers in their role as motivators tend to have high TX levels.

**FIGURE 6:**
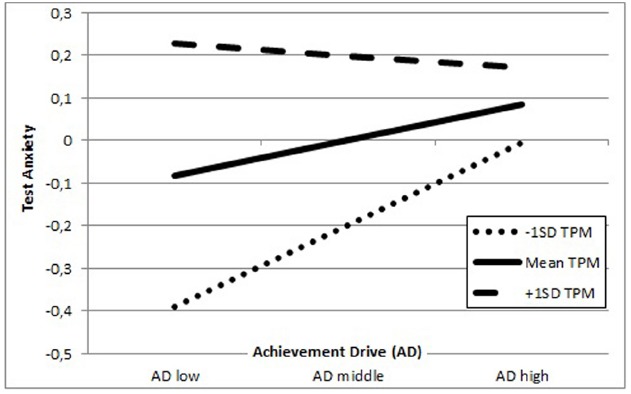
**Representation of teachers as positive motivators (TPM) as moderator in the association between achievement drive (AD) and test anxiety (TX) among Canadian secondary school students; the Y-axis shows test anxiety and the X-axis shows achievement drive**.

As depicted in Figure 6, students scoring low on AD and low on TPM also report low on TX. Whereas, students who report low AD while relying on their teachers as a source of motivation tend to have higher levels of TX. With increasing AD the reported levels of TX increase for students with average and below average TPM. In contrast, levels of TX do not increase with higher AD for students with a high level of TPM. There is a trend toward lower TX with increasing AD for this subgroup of students. In summary, the tendency of increasing levels of TX with increasing AD (see Figure 5, for the direct effect) is neutralized and to some extend reversed by a perception of teachers acting as positive motivators, thus displaying nicely the buffering effect of TPM.

**FIGURE 7:**
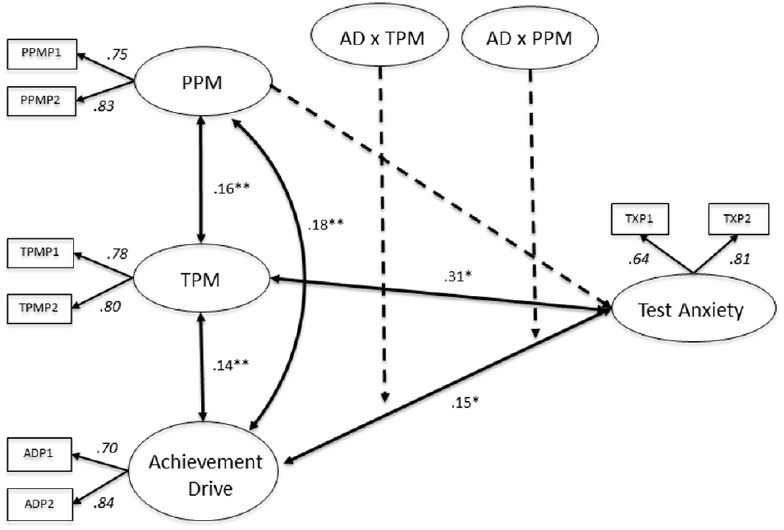
**Multigroup latent moderated structural equations (MGLMS) for the German sample.** Significant effects shown as unstandardized coefficients (B), bold pathways are significant at *p < 0.05, **p < 0.001; dotted pathways are not significant. Factor loadings are standardized (based on the standardized results of the weak measurement model seen as the final model only reports unstandardized results). PPM, peers as positive motivators; TPM, teachers as positive motivators; AD, achievement drive.

Among German students, neither TPM nor PPM were found to moderate the association between AD and TX (Figure 7). Direct effects of the MGLMS 2 reveal a significant relationship between AD and TX (*B* = 0.15, SE = 0.05, *p* < 0.05). Similar to Canadian students, the more students are oriented toward achievement, the more they report of TX. Furthermore, there is a significant association between TPM and TX (*B* = 0.31, SE = 0.10, *p* < 0.05), indicating that students who depend on teachers in their role as motivators tend to have high TX levels.

## Discussion

The current study examined the relationship between AD and TX among Canadian and German secondary school students. Moreover, it explored the potential of socio-motivational relationships, such as SSR, TSR, PPM, and TPM, in buffering the association of AD and TX. To our knowledge, no such study was implemented to date. To test our original hypotheses, two MGLMS were examined.

### The Buffering Role of Student–Student and Teacher–Student Relationships Among Canadian and German Secondary School Students

The first hypothesis was partially confirmed since SSR significantly moderated the relationship between AD and TX for Canadian students and TSR acted as moderator among German secondary school students. However, TSR did not moderate this association among Canadian students and SSR were not found to act as moderator among German students.

Canadian students who experienced high quality SSR, hence perceived the class environment as inclusive and supportive tended to report the lowest TX levels in comparison to students with average or below average SSR. Various studies have found that peers help students orient themselves along mutual educational expectations and trajectories, values, behavior as well as school adjustment (Hussong, 2002; Espelage et al., 2003; Kiuru et al., 2007; McGloin et al., 2014), while reciprocal enforcement and socialization takes place (Harris, 1995; Shin and Ryan, 2014). The prospect of gaining prestigious status is given in case peer values are being followed and certain behavior is being demonstrated (Rubin et al., 2006). Hence, peers’ academic success is an incentive for students to boost their academic performance (Kaufmann, 2007). In fact, with increasing levels of AD, students who felt part of a peer groups continued to report low TX levels as found among Canadian students. Hence, they perceived their SSR as protector against feelings of TX. In fact, students perceiving high quality peer relationships showed increased well-being, self-esteem, and decreased levels of TX (cf. Leary and Baumeister, 2000; Arnett, 2007; Flanagan et al., 2008; Hoferichter and Raufelder, 2014).

Canadian students who simultaneously experienced low AD and poor quality SSR reported high levels of TX. These students might not be as engaged in school and learning activities with their classmates as they may have different priorities other than to excel academically and to engage in social relationships with classmates. With increasing levels of AD, TX levels tended to decrease, however still being higher than of students with high quality SSR. It can be assumed that the student group reporting high achievement orientation and low quality SSR is more confident about their performance, hence showing lower TX scores than students with low AD and low quality peer relationships.

German students who reported low AD and simultaneously perceived a high quality relationship with their teachers, tended to report a high degree of TX in comparison to students with low AD and low quality TSR. However, even for the first mentioned student type the TX levels appeared to be below average. Students might feel that they have to fulfill teacher’s expectations, which in turn lead to higher levels of TX. In fact, research has indicated the existent link between expectations set by significant others and high levels of TX as well as low school performance (cf. Zeidner, 1998; Zohar, 1998; Gill and Reynolds, 1999). Students who perceive their relationships as obligatory under a stressful event do not benefit from these relationships (Bolger and Eckenrode, 1991). Furthermore, German students also reported increasing TX levels with increasing AD, irrespective of their relationship quality with teachers. This result was confirmed by the significant positive association between AD and TX. Numerous research indicates that the desire to achieve is accompanied by the fear of failure (Hodapp and Benson, 1997; Zeidner, 1998; Elliot and McGregor, 1999) and pressure to perform (cf. Atkinson, 1974; Singh, 2011), while in turn the latter represent the main stressors in daily school life (Seiffge-Krenke, 1995).

In summary, SSR buffered feelings of TX related to AD among Canadian students, while for German students their TSR acted as moderator but intensified feelings of TX. Canadian teachers see the provision of teacher and peer support for students’ academic and personal development as well as the development of moral and civil values as constituting an integral part of their teaching profession (Hesse, 2004). In contrast, the TSR for German secondary school students can be described as rather impersonal (Hesse, 2004) and is characterized by an imbalance of power between students and teachers (Raufelder, 2007). This could explain why achievement oriented students with ranging quality of TSR tend to report high TX levels, while no pattern of mitigation deriving from TSR could be found.

### The Buffering Role of Teachers as Positive Motivators Among Canadian Secondary School Students

The second hypothesis was also partially confirmed as TPM moderated the association between AD and TX among Canadian secondary school students only. Students with low AD who turned to their teachers for motivation, experienced higher TX levels compared to low achievement oriented students who did not rely on their teachers for motivation. Hence, students that gain their motivation from their relationships with teachers may feel pressured to meet teachers’ expectations in test situations (Hoferichter et al., 2014a). Further research indicates that students who mainly depend on teachers to feel motivated tend to report higher TX as well as negative social interactions, including behavioral problems at school (Pianta and Nimetz, 1991; Hamre and Pianta, 2001; Sabol and Pianta, 2012; Hoferichter and Raufelder, 2014). Other students who are neither eager to excel at school tasks nor do they turn to their teachers for their own motivation, are less test anxious. Seen as the prospect of attaining academic success and being able to relate to the teacher might not appeal to them, they might not be all that sensitive to scholastic matters in general. For students relying on their teachers to motivate them, levels of TX slightly decreased with increasing AD. In this case it is students’ relationships with their teachers that motivate them in school and thus buffer the effect of AD on TX. In addition to these findings, Williams (1980) found that high achieving students tend to identify with their teachers more than low achieving students. Seen as these students are eager to succeed, their motivating relationship with teachers dampens the development of TX. This finding supports the buffering hypothesis, according to which social relationships protect individuals from the detrimental effects of stressful situations, which in the current study are pressure to perform and the avoidance of failure as components of AD (Cohen and Wills, 1985; Stravynski and Boyer, 2001; Adams et al., 2011; Johnson et al., 2011). In fact, high achieving students who lack a motivating relationship with their teachers report higher TX with increasing AD as they do not profit from their teachers in this respect.

Further research would be well advised to evaluate other related aspects that might add to this model. For example, personality aspects such as neuroticism have been shown to be related to high anxiety levels (Chamorro-Premuzic et al., 2008) and TX in particular (Spielberger and Vagg, 1995; Fitch, 2004; Hoferichter and Raufelder, 2013), while conscientiousness and agreeableness are predictors of close and non-conflictual relationships (Zee et al., 2013). Furthermore, social exclusion and feelings of isolation and loneliness are all factors that might contribute to the development of TX (Lee and Robbins, 1998; Gaspar de Matos et al., 2003; Bukowski et al., 2010).

## Conclusion

In summary, this study reveals the qualitative impact of socio-motivational relationships on secondary school students’ AD and TX measured in Canada and Germany. This study provides a model of key interactions in the school context that work to support academic performances and alleviate negative outcomes such as TX. In particular, this study reveals how TSR, SSR, and TPM can lead to contradictory outcomes depending on students’ attitude toward AD. Moreover, these variables and their respective effects may vary across nations, in this case Canada and Germany. The contradicting buffering effects of socio-motivational relationships present themselves as two sides of the same coin and contribute to cross-national research. For instance, in the German sample high quality TSR do not protect against TX among achievement oriented students but contribute to a further increase of TX. Yet in Canada, teachers perceived as positive motivators protect achievement oriented students from feeling test anxious. Additionally, among Canadian students, SSR buffer feelings of TX.

The results of this study may be of use to teachers and educators by helping them alleviate students’ TX through careful consideration of individual differences and increased awareness of group dynamics and team spirit in school, which are founded on socio-motivational relationships. This study aims to disentangle the relationship between AD and TX by considering the role of socio-motivational relationships among Canadian and German secondary school students. Its results address the alarming trend observed by Schab (1991) among students of the Western hemisphere: students mention fear of failure as being the most common reason for cheating on tests and homework, while viewing dishonesty as a necessity and attributing successful career paths to fraudulent activities. In the long term, the promotion of competition and egocentric activities as opposed to cooperation with peers seems to remain a dominant trend in contemporary educational models. McClelland (1961) already identified this trend in his often cited work on the “Achieving Society,” which has not lost its validity in the 50 years since its original publication (McClelland, 2010).

The trend to surpass competitors by all means may be deeply rooted in contemporary Western societies and might have contributed to the general increment of school-related anxiety observed over the last decades (Spielberger and Rickman, 1990; Twenge, 2000). To counteract this trend, socio-motivational relationships may buffer TX related to AD as observed in the case of Canadian students who are oriented toward teachers acting as motivators as well as for those who report high quality SSR. With the variables studied, the buffering role of socio-motivational relationships for German students could not be established as it was established for Canadian students, because German students, perceiving high and low quality TSR displayed increasing TX levels with increasing AD. No moderator has been found in Germany to mitigate feelings of TX related to AD.

### Strengths, Limitations, and Future Directions

This study compares the buffering effect of socio-motivational relationships in the association between AD and TX among Canadian and German students. By disclosing both possible buffering outcomes, i.e., intensifying or dampening TX that emerges from AD, the findings contribute to the literature and provide practical implications for schools. The buffering hypothesis proposed by Cohen and Wills (1985), which assumes that social relationships generally mitigate stressful situations, is hereby extended. The current study reveals the dual effect of socio-motivational relationships in school contexts, which function as buffers in the interplay of AD and TX, varying across Canada and Germany. This study follows a contextual and cross-national approach, which has rarely been used in the disciplines of education and psychology (cf. Markus and Kitayama, 1991; Bodas and Ollendick, 2005; Shernoff and Schmidt, 2008), by acknowledging socio-motivational relationships as potential buffers among Canadian and German students. Readers should be aware of the current study’s limitation when interpreting its results. The study applies to adolescent students between 12 and 16 years of age from Quebéc and Brandenburg, who commented on their socio-motivational relationships with their teachers and peers, their drive for achievement, and TX. Due to the cross-sectional nature of our data, causal effects must not be derived from the results provided.

With our study, we hope to invoke further contextual and cross-national studies, as we believe this approach to be particularly fruitful to the field of education and psychology. Prospective studies may build on this study by including different age groups (e.g., elementary students) from various countries (e.g., China, South Africa, Russia, etc.) and therefore accounting for different educational systems, informed by many sources (reports from teachers, peers, parents, etc.), and following a longitudinal design with multiple measurement points over time.

### Conflict of Interest Statement

The authors declare that the research was conducted in the absence of any commercial or financial relationships that could be construed as a potential conflict of interest.
